# Gustatory sweating in people with type 1 and type 2 diabetes mellitus: Prevalence and risk factors

**DOI:** 10.1002/edm2.290

**Published:** 2021-08-10

**Authors:** Carina Kirstine Klarskov, Elena von Rohden, Birger Thorsteinsson, Lise Tarnow, Peter Lommer Kristensen

**Affiliations:** ^1^ Department of Endocrinology and Nephrology Nordsjællands Hospital Hillerød Denmark; ^2^ Department of Clinical Medicine Faculty of Health and Medical Sciences University of Copenhagen Copenhagen Denmark; ^3^ Steno Diabetes Center Sjælland Holbæk Denmark

**Keywords:** gustatory hyperhidrosis, gustatory sweating, nephropathy, neuropathy, retinopathy, type 1 diabetes, type 2 diabetes

## Abstract

**Objective:**

Gustatory sweating (GS) is characterized by profuse sweating during or immediately after ingestion of food and is known as a complication of diabetes mellitus (DM). This study aimed to determine the prevalence of GS and to characterize the sweating in a cohort of patients with type 1 and type 2 diabetes mellitus (T1DM and T2DM) as compared with a control group.

**Methods:**

In a cross‐sectional study, 665 outpatients with T1DM and 505 outpatients with T2DM filled in an 8‐point questionnaire about GS. Answers were paired with medical data from the electronic patient records to explore associations with DM complications. The control group consisted of 1158 persons without DM answering the same questionnaire in an online version.

**Results:**

In people with T1DM and T2DM, the prevalence of GS was 10% (95% CI 7%–12%) and 13% (95% CI 10%–16%), respectively. In the control group, the prevalence of GS was 5% (95% CI 3%–6%). Most commonly, people sweat on the face and/or head and upper body with a duration of 10–30 min albeit in the control group <10 min. In patients with T1DM, increased HbA1c was associated with GS (OR 1.3 [95% CI 1.05–1.6], *p* = .016), and in T2DM, younger age (OR 0.95 [95% CI 0.92–0.99), *p* = .006), presence of severe peripheral neuropathy (OR 2.33 [95% CI 1.04–5.2], *p* = .039) and absence of proliferative retinopathy were associated with GS (OR 0.22 [95% CI 0.07–0.71], *p* = .011).

**Conclusion:**

We found the prevalence of gustatory sweating of 11% in a hospital‐based cohort of patients with T1DM and T2DM. This was twice as high as in non‐diabetic control persons. Associations between GS and known diabetes complications could only be demonstrated in T2DM. Compared with a control group without DM, odds for GS are higher in people with DM and age >45.

## Graphical abstract

Gustatory sweating (GS) is characterized by profuse sweating during or immediately after ingestion of food and is known as a complication of diabetes mellitus (DM). In a cross‐sectional study, 665 outpatients with T1DM and 505 outpatients with T2DM and 1158 controls filled in an 8‐point questionnaire about GS. The overall prevalence of gustatory sweating of around 11% was found in a large unselected hospital‐based cohort of patients with T1DM and T2DM, which is approximately twice as high as in non‐diabetic control persons.

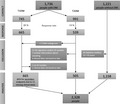

## 1 INTRODUCTION

Gustatory sweating (GS) is a secondary form of focal hyperhidrosis triggered by food intake. GS can either be physiological such as when eating spicy foods or be non‐physiological where the response is independent of food type.^1^ There is no internationally agreed definition of GS. In a position statement from the American Diabetes Association (ADA) on diabetic neuropathy, GS is described as a sudomotor dysfunction with profuse sweating on the face and neck in relation to food intake (or in some cases the smell of food).^2^ In the current study, we have expanded the definition to include sweating also from other parts of the body in relation to food intake. Although considered a harmless condition, GS can cause distress and strong feelings of shame. Many patients report withdrawal from eating in social settings, which has a strong negative impact on their quality of life.^3^, ^4^, ^5^ In some cases, GS disrupts normal eating patterns, which can lead to poorer glycaemic control and potentially life‐threatening hypoglycaemia in patients with insulin‐treated or sulphonylurea‐treated diabetes (DM).^6^, ^7^

The pathophysiology behind GS in diabetes is unknown. Some studies hypothesize that it is a manifestation of autonomic dysfunction due to aberrant nerve fibre regeneration.^4^, ^5^, ^8^ Others discuss evidence of separate aetiologies like compensatory thermoregulation, anti‐sympathetic ganglia antibodies, neuropathic loss of suppression of nerval tonus that controls sweating and the role of reversible molecular changes due to nephropathy.^9^, ^10^, ^11^ The distribution of sweating is equivalent to the territory of the superior cervical ganglion^5^ and can be objectified, by applying quinizarin powder to the face, head and upper torso/extremities (turning blue when getting wet)^5^or by weighing absorbent dressings worn during meals.^3^ Both methods are impractical in larger cohorts.^3^ As an alternative, Shaw et al^10^ used a questionnaire for self‐reported GS. To test the reliability of the questionnaire, they made 25 random samples and found that it was a reliable way of reporting GS.^10^

The prevalence of a gustatory sweat response in people with DM compared to the background population has not yet been established. Neither has its potential relation to other DM complications been investigated.

In this study, we assessed the prevalence of GS in cohorts of people with type 1 and type 2 DM (T1DM and T2DM) and in a control group without DM. Furthermore, we describe duration and body location of GS during meals, and to generate hypotheses on the pathophysiology of GS we looked for associations between GS and late diabetic complications as well as different indices of glycaemic control.

## 2 SUBJECTS, MATERIAL AND METHODS

### 2.1 Design

**FIGURE 1:**
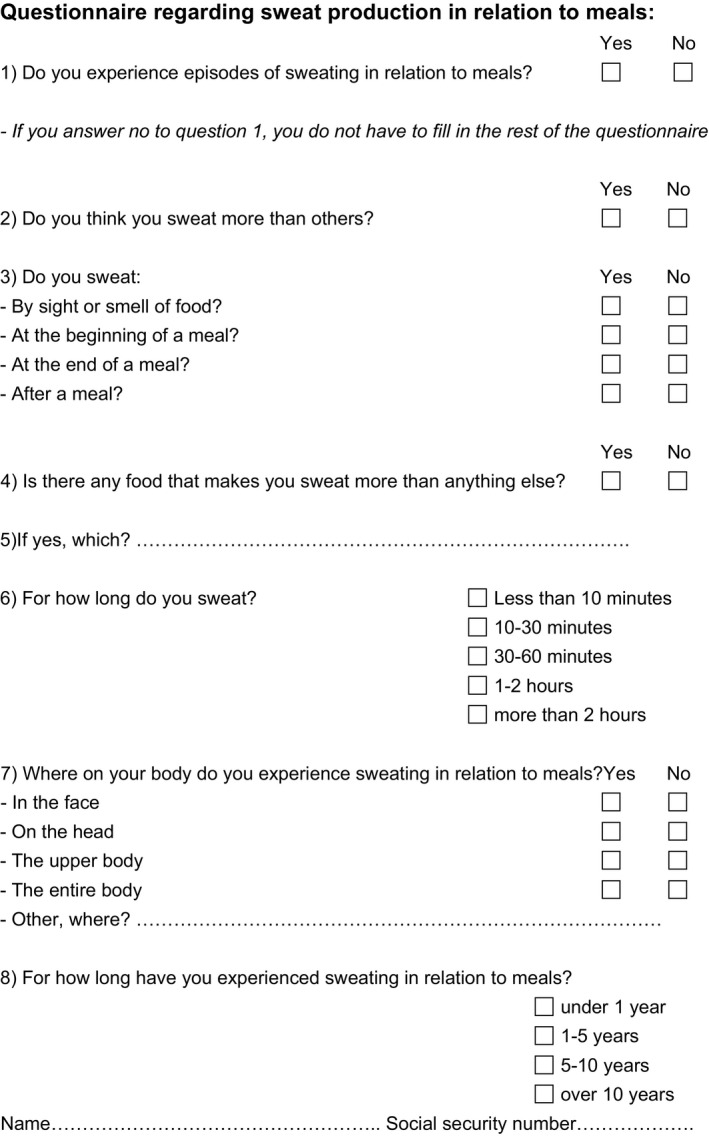
Questionnaire sent to 745 patients with type 1 diabetes from Steno Diabetes Center and 991 patients with type 2 diabetes from Nordsjællands Hospital. A total of 1158 controls completed the exact same questionnaire in an online form

In a cross‐sectional study, 745 outpatients with T1DM at Steno Diabetes Center (now Steno Diabetes Center Copenhagen), Denmark, and 991 outpatients with T2DM in the diabetes clinic at Nordsjællands Hospital, Denmark, received a questionnaire by mail with eight questions regarding GS (Figure 1). Answers were paired with medical data from the electronic patient records. The study was approved by the Danish Data Protection Agency (#2012‐58‐0004). Patients were informed about the scope, purpose and design of the study in an accompanying letter and gave consent to use previously collected clinical data by sending back a signed filled‐in questionnaire.

Inclusion criteria were a diagnosis of DM based on the diagnoses (ICD‐10 diagnosis code: DE10.X = T1DM and DE11.X = T2DM) recorded in the electronic patient record, and age >16 years.

Exclusion criteria were questionnaires received too late, a lack of ID‐number or signature.

A control group of 1158 people with no prior or current diagnosis of diabetes answered the same 8‐point questionnaire in an online version distributed through social media. The questionnaire was made available to an unselected cohort on several social platforms. Exclusion criteria (age below 16 years or yes to current or prior DM) were asked as the first questions, and the GS questionnaire only opened if no exclusion criteria were met.

### 2.2 Clinical data collection

To test for association between the presence of GS and diabetic complications, clinical data from patient records were collected as close to the date of the return of the questionnaire as possible and at least within a year. The following data were collected: duration of diabetes (years), HbA1c (mmol/mol), peripheral neuropathy (assessed by biothesiometry and defined as present if vibration perception threshold (VPT) was ≥50 V on one foot), nephropathy (urinary albumin/creatinine‐ratio [UACR] subdivided into normoalbuminuria [<30 mg/g], microalbuminuria [30–300 mg/g] and macroalbuminuria [>300 mg/g]), diabetic retinopathy (classified in none, non‐proliferative diabetic retinopathy [NPDR] and proliferative retinopathy based on digital fundus photography). In the control group, age, sex, height, weight and yes/no to kidney disease were self‐reported.

### 2.3 Primary endpoint

The prevalence of GS was the primary end‐point. GS was defined as present if a patient answered yes in box 1 and 2 in the questionnaire (Figure 1). The cohort with GS was further split up into physiological and true GS. A sweat response was considered physiological if any known spicy foods were reported in question 5. True GS was defined as sweating not only triggered by spicy foods. Patients that mentioned inconclusive GS food triggers were excluded from the true GS group.

### 2.4 Secondary endpoints

The other items on the questionnaire (duration, location, time of sweating regarding meals and start of GS symptoms) were secondary end‐points used to further characterize the sweating. The true GS and physiological GS groups were compared. Correlations between known diabetic complications and the risk of GS were additional explorative end‐points. Gender, age, duration of DM, HbA1c, nephropathy, retinopathy and neuropathy were included in the analysis.

### 2.5 Statistical analyses

Data are presented for T1DM, T2DM and the control group separately. All numerical data were assessed for normality by using a one‐sample Kolmogorov‐Smirnov test, which indicated that all data were normally distributed, except for age at diagnosis of DM. Consequently, a Mann‐Whitney *U* test was performed for this variable. Comparing other continuous variables was done using independent Welsh *t* tests. Continuous data are presented as means (±1SD). Categorical data were compared with a chi‐square test and are presented as percentage.

To identify variables associated with GS, the following variables were included: sex, age, duration of diabetes, HbA1c, albuminuria, retinopathy and neuropathy. A multiple logistic regression was performed to model the interdependent influence of these variables on risk of GS, and odds ratios were calculated. To minimize collinearity between variables in the multiple logistic regression analysis, duration of DM was included and age at DM diagnosis was excluded. A two‐tailed *p*‐value of ≤.05 was considered statistically significant. All statistical analyses were performed using SPSS 25.0 (SPSS).

To control for overall age differences in the DM cohort and the control group, five age groups were defined as 16–29, 30–44, 45–59, 60–74 and 75+ years of age. Moreover, to clarify whether perimenopausal sweating in woman contributes to the prevalence of GS, we did a supplementary analysis comparing prevalence of GS in men and women aged 40–60 years in both the DM group and in the control group.

## 3 RESULTS

**FIGURE 2:**
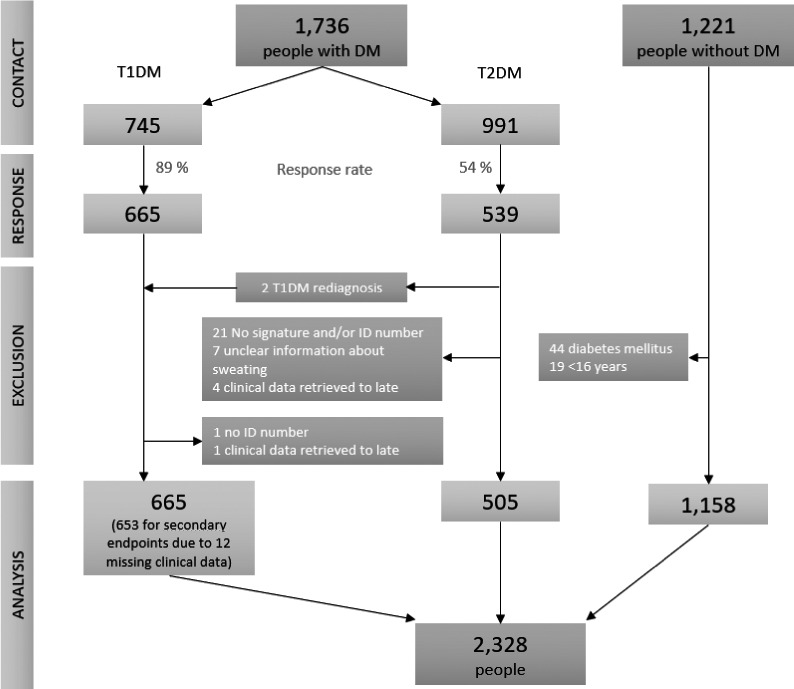
Response rate and selection of final study population. Two persons were re‐diagnosed with T1DM and included in the T1DM cohort

Questionnaires were sent out to totally 1736 people with T1DM and T2DM and 1204 valid questionnaires (69%) were returned (Figure 2). For people with diabetes, the questionnaires were sent and returned from December 2016 to January 2017 and in the control group, the survey was conducted from January 2021 to February 2021. A total of 665 of 745 patients with T1DM responded resulting in a response rate of 89%. In patients with T2DM, 539 of 991 patients answered, resulting in a response rate of 54%; however, 34 questionnaires were excluded from the primary analysis due to the exclusion criteria. The baseline characteristics for the true GS and non‐GS groups are listed in Table 1. In the control group 1158 people aged 16 years or more without DM completed the questionnaire. 44 were excluded due to self‐reported DM and 19 due to age under 16 years. It was not possible to calculate a response rate for the control group due to the use of social media to distribute the questionnaire.

**TABLE 1:** Clinical characteristics of 2328 people, 653 with type 1 diabetes, 505 with type 2 diabetes and 1158 without diabetes—with and without gustatory sweating

Variable	Type 1 diabetes			Type 2 diabetes			Control group		
Total *n* = 2328	GS (*n* = 64)	No GS (*n* = 589)	*p*	GS (*n* = 65)	No GS (*n* = 440)	*p*	GS (*n* = 54)	No GS (*n* = 1104)	*p*
Male sex, %	45	50	.501	59	70	.113	33	35	.768
Age, years (median; range)	52 ± 14 (51;17–86)	52 ± 15 (52;16–90)	.767	64 ± 12 (65;36–88)	69 ± 10 (69; 26–91)	.**008**	52 ± 13 (54; 20–74)	52 ± 14 (52;16–82)	.839
Age at DM diagnosis, years	28 ± 17	25 ± 15	.150	51 ± 11	55 ± 12	.**044**	—	—	—
Duration of DM, years (median; range*)*	23 ± 14 (25.5;1–54)	27 ± 14 (28;0–70)	.182	14 ± 9 [10] (13;0–43)	14 ± 9 [3] (13;0–51)	.888	—	—	—
Weight, kg	76.1 ± 15	76.8 ± 15	.703	93 ± 18.9 [10]	92.3 ± 20.7 [5]	.793	89 ± 22	81 ± 18	.**001**
Blood pressure, mmHg	137/78 ± 18/11 [1]	135/77 ± 17/10 [5]	.265/.428	135/78 ± 14/9 [11]	138/76 ± 16/10 [2]	.17/.15	—	—	—
HbA1c, % (mmol/mol)	8.4 ± 1.3 (68 ±14)	8.0 ± 1.1 (64 ±12)	.**007**	7.9 ± 1.5 (63 ± 16) [10]	7.9 ± 1.4 (63 ± 15) [4]	.973	—	—	—
U‐albumin/Creatinine‐ratio	208 ± 1010	64 ± 272	.361	134 ± 396	258 ± 717	.252	—	—	—
Nephropathy, %			.784	[18]	[88]	.213			
Normoalbuminuria	69	73		57	49		—	—	—
Microalbuminuria	27	23		36	36				
Macroalbuminuria	5	5		6	16				
Retinopathy, %				[15]	[85]	.076			
None	—	—		67	56		—	—	—
Non‐proliferative	—	—		20	18				
Proliferative	—	—		13	27				
Neuropathy (VPT ≥ 50 V), %	11 [2]	7 [25]	.285	3 [13]	34 [18]	.785	—	—	—
Self‐reported Kidney Disease (Control group) %	—	—	—	—	—	—	0	0.9	.483

Note

Statistically significant *p*‐values are bold. Categorical data are presented in %, numerical data in mean ± 1SD. Number of missing (*n*) values are shown in square brackets []. No number in brackets indicates that this variable had no missing values.

Abbreviations: DM, Diabetes Mellitus; GS, Gustatory Sweating; V, Volt; VPT, Vibration Perception Threshold.

### 3.1 Primary endpoint

A total of 1170 people, 665 with T1DM and 505 with T2DM, were included in the analysis of the primary endpoint. In the T1DM cohort, 13% of the patients had physiological GS (they answered yes to question 1 and 2) and 10% (95% CI 7%–12%) had true GS. In the T2DM cohort, 22% of the patients had physiological GS and 13% (95% CI 10%–16%) had true GS. In the control group, 1158 people were included, 9% had physiological GS and the prevalence of true GS was 5% (95% CI 3%–6%).

### 3.2 Secondary endpoints

The analysis of the questionnaire data regarding the sweat characteristics used data of all 1170 people with DM, but for the logistic regression analysis, only data of 1158 people (653 with T1DM and 505 with T2DM) were analysed because clinical data were missing in 12 patients with T1DM.

#### 3.2.1 Comparison of GS and non‐GS groups

People with T2DM and GS were statistically significantly younger than people without GS (+GS: mean ± SD 64 ± 12 years vs −GS 69 ± 10, *p* = .008), whereas people with T1DM with and without GS had similar mean ages (Table 1). People with T1DM and GS had a statistically significantly higher HbA1c than people without GS (+GS: 68 ± 14 mmol/mol vs −GS: 64 ± 12, *p* = .007), whereas people with T2DM with and without GS had similar mean HbA1c. Distribution of different stages of nephropathy and neuropathy was not different in the GS and non‐GS group, either for T1DM or T2DM. For people with T1DM, the obtainable clinical data about stage of retinopathy were not conclusive, and therefore, this variable was excluded from further analysis. In the control group, people with GS had significantly higher weight than people without GS (+GS: 89 ± 22 kg vs −GS: 81 ± 18 kg, *p* = .001) (Table 1).

#### 3.2.2 Characteristics of sweating

The debut of GS, bodily distribution of sweating, triggers and duration for both T1DM, T2DM and the control group are presented in Table 2.

**TABLE 2:** Reported start of gustatory sweating as well as locations, triggers and duration of sweating in people with gustatory sweating and type 1 diabetes, type 2 diabetes or in a control group

	Type 1 diabetes	Type 2 diabetes	Control group
	*n* = 64	*n* = 79	*n* = 54
Start of GS symptoms			
>1 year	14 (22%)	15 (19%)	6 (11%)
1–5	28 (44%)	38 (48%)	15 (28%)
5–10	7 (11%)	13 (17%)	13 (24%)
>10 years	13 (20%)	13 (17%)	20 (37%)
Location			
Face	39 (61%)	49 (62%)	44 (82%)
Head	19 (30%)	52 (66%)	35 (65%)
Upper body	41 (64%)	43 (54%)	37 (69%)
Entire body	8 (13%)	11 (14%)	20 (37%)
Trigger			
Sight or smell of food	4 (6%)	10 (13%)	3 (6%)
Beginning of a meal	20 (31%)	24 (30%)	9 (17%)
End of a meal	36 (56%)	49 (62%)	34 (63%)
After a meal	30 (47%)	59 (75%)	42 (78%)
Duration of sweating			
<10 min	22 (34%)	23 (29%)	24 (44%)
10–30 min	37 (58%)	29 (37%)	21 (39%)
30–60 min	3 (5%)	18 (23%)	6 (11%)
1–2 h	1 (2%)	4 (5%)	3 (6%)
>2 h	—	6 (8%)	—

Note

Numbers are absolute values (per cent). Multiple answers were possible. Other self‐reported locations of sweating not shown in Table 5 were back of the neck (6), axilla (5), forehead (2), under the breasts (2), lower back (2), legs (1), feet (3), chest (1), right wrist (1), neck (1), hands (2), hairline (1) and back of the knees (1) (number in brackets represents number of people reporting the location).

Most people with DM had a history of GS symptoms for 1–5 years (44% in T1DM and 48% in T2DM) but in the control group, most people had GS symptoms for >10 years (37%). Some patients, however, report having experienced the symptoms for less than 1 year and for more than 10 years.

All patients sweat mainly in the head and upper body, and very few people with DM sweat on the entire body. People with T1DM tend to sweat more in the face and upper body, whereas patients with T2DM have an equal distribution of sweating in the face, head and upper body. People in the control group sweat more often on the entire body than the DM groups.

In most people, GS starts by the end of or after a meal. Most people with T1DM and T2DM respondents reported duration of sweating to be 10–30 min (58% in T1DM and 37% in T2DM). In the control group, the most reported duration was <10 min (44%). There was a tendency for people with T2DM to sweat longer than both people with T1DM and the control group (T2DM 23%, T1DM5% and control group 11% sweat for 30–60 min, respectively).

The non‐spicy trigger foods for GS that were mentioned by more than one patient were soup and fruit in T1DM, fatty foods and meat or beef in T2DM, and candy in the control group. Foods that were mentioned by both patients with T1DM, T2DM and in the control group as triggers were soups, fatty foods, sugary foods and cheese.

#### 3.2.3 Explorative analysis of associations between gustatory sweating and DM complications

In patients with T1DM, logistic regression analysis showed that increasing HbA1c was associated with increasing probability of GS (OR 1.3 [95% CI 1.05–1.6], *p* = .016), Table 3. In patients with T2DM, logistic regression analysis showed an association between low age and probability of GS (OR 0.95 [95% CI 0.92–0.99], *p* = .006). Furthermore, the presence of severe peripheral neuropathy (threshold of biothesiometry ≥50 V) (OR 2.33 [95% CI 1.04–5.2], *p* = .039) and the absence of proliferative retinopathy were associated with higher risk of GS in T2DM (OR 0.22 [95% CI 0.07–0.71], *p* = .011).

**TABLE 3:** Multiple regression analyses of risk of gustatory sweating in 653 patients with type 1 diabetes mellitus and in 505 patients with type 2 diabetes mellitus

Variable	Odds ratio					
	Type 1 diabetes			Type 2 diabetes		
	OR	95% CI	*p*	OR	95% CI	*p*
Male sex	0.82	0.48–1.42	.374	0.55	0.25–1.2	.134
Age	1.01	0.99–1.03	.318	0.95	0.92–0.99	.**006**
Duration of DM	0.98	0.96–1.0	.093	1.03	0.98–1.07	.262
HbA1c (mmol/mol)	1.3	1.05–1.6	.**016**	1.01	0.98–1.03	.650
Nephropathy						
Normoalbuminuria	1 (reference)					
Microalbuminuria	1.1	0.58–2.14	.758	1.1	0.52–2.34	.807
Macroalbuminuria	1.2	0.32–4.34	.799	0.5	0.13–1.91	.312
Retinopathy						
None	1 (reference)					
Non‐proliferative	—		—	0.83	0.33–2.12	.697
Proliferative	—		—	0.22	0.07–0.71	.**011**
Neuropathy						
VPT <50 V	1 (reference)					
VPT ≥50 V	1.63	0.64–4.16	.309	2.33	1.04–5.2	.**039**

Abbreviations: DM, Diabetes Mellitus; OR, Odds Ratio; V, Volt; VPT, Vibration Perception Threshold.

#### 3.2.4 Influence of age on the prevalence of GS

We considered the possible influence of age on the prevalence of GS and calculated the odds ratio of true GS in five age groups for the cohort of people with DM compared with the control group without DM. For age groups 1 and 2, there was no statistically significant difference between the DM groups and the control group for true GS. For age group 3, 4 and 5, people with DM had statistically significantly increased odds of true GS: 45–59 years: 3.75 (95% CI 2.14–6.58), *p* < .001, 60–74 years: 1.95 (95% CI 1.15–3.31), *p* = .015 and 75+ years: 4.02 (95% CI 1.24–4.13), *p* = .009 (Table S1).

#### 3.2.5 Menopause

To clarify whether perimenopausal sweating in woman contributed to the prevalence of GS, a supplementary analysis of the prevalence of GS in men and woman aged 40–60 years was carried out. Neither in the DM groups nor in the control group showed differences between sexes in both groups (odds ratio true GS for men compared with women was 0.77 [95% CI 0.49–1.22], *p* = .295). This indicates that perimenopausal sweating in woman probably did not bias our results.

## 4 DISCUSSION

We found the overall prevalence of GS of around 11% in a large, hospital‐based cohort of patients with T1DM and T2DM. GS was associated with higher HbA1c in T1DM and with lower age, severe peripheral neuropathy and absence of proliferative retinopathy in T2DM. In the control group, we found the overall prevalence of GS of 5%. When comparing in age groups, we found that people with DM and age above 45 years had increased risk of true GS. To our knowledge, this is the first study to look at the prevalence of GS in both people with DM and without DM. The results support the general view that GS is a complication to DM.

Even though GS caused by Frey's syndrome or surgical complications has been mentioned as early as 1757,^12^, ^13^, ^14^ the first cases of GS in diabetes were published by Watkins et al^5^ in 1973. Since then, the phenomenon has mainly been described in case reports^4^, ^7^, ^15^, ^16^, ^17^, ^18^, ^19^, ^20^, ^21^ and has been referred to as a common symptom of diabetic autonomic neuropathy in a study by Shaw et al.^10^

Watkins et al investigated GS in six patients with T1DM and diabetic complications such as diabetic diarrhoea, impotence, retinopathy and proteinuria by collecting clinical data, sweat tests and atropine administration.^5^, ^10^ GS was demonstrated by using quinizarin powder, and the sweating pattern was distributed to head, neck, shoulders and upper part of the chest. It was provoked by chewing specific trigger foods, particularly cheese, and in one patient by the thought of food alone.^5^ The distribution of GS correlating to the territory of the superior cervical ganglion made the authors suggest that GS is due to abnormal regrowth of damaged vagal nerve ends to sympathetic cholinergic sweat fibres at the level of the superior ganglion. When atropine was administered to three patients, symptoms of GS disappeared, supporting their hypothesis.^5^

We were able to confirm the distribution of sweating to mainly head, face and upper body through a self‐reported questionnaire in both people with diabetes and the control group. The trigger foods mentioned in previous studies, such as cheese or chocolate, could not be confirmed as strong triggers (albeit mentioned by a few patients) in our study but patients mentioned a wide range of trigger foods with fatty foods, meat/beef and soup being mentioned most often.^5^, ^11^ The similarity of characteristics of GS in people with physiological and non‐physiological sweating supports the idea of similar pathways as the physiological sweat response to spicy food. We found that duration of sweating in connection to a meal is often long, for some patients (with type 2 diabetes) up to 2 h and that GS for some patients is a long‐standing complication being present for years.

In a study from 1996 using patient interviews and including 152 subjects with T1DM or T2DM and 44 without DM, Shaw et al showed a significant independent association between DM and GS.^5^, ^10^ They found that GS occurred in 69% of patients with diabetic nephropathy (*n* = 59), and in 36% of patients with diabetic neuropathy, which was tested peripherally as VPT with a neurothesiometer (*n* = 42). The control groups, one group of T1DM and T2DM without nephropathy or neuropathy (*n* = 51) and one group with non‐diabetic nephropathy (*n* = 44), had the prevalence of GS of 4% and 2%, respectively. Shaw et al^10^ also showed an association between GS and neuropathy, younger age and urinary protein excretion in cohorts including both patients with T1DM and T2DM. Shaw et al and our study both found an association between neuropathy and GS, supporting the hypothesis of GS being a neurological manifestation. Shaw et al proposed a strong link between GS and reversible molecular changes due to diabetic nephropathy because they observed the cessation of GS symptoms in five patients as soon as 48 h after kidney transplantation, and the high prevalence in patients with diabetic nephropathy. In contrast, we did not find any association between any degree of nephropathy and GS. The association between GS and younger age that Shaw et al found was confirmed in the T2DM group in our study. Unfortunately, we did not have access to data on autonomic dysfunction in the present study.

The positive association between high HbA1c and GS in T1DM has not been reported in previous studies but two case reports describe this connection. Van der Linden et al describe a 39‐year‐old woman with T1DM for the past 37 years, persistently uncontrolled blood glucose levels and an HbA1c of 72 mmol/mol who experienced severe GS.^11^ In another case, a 44‐year‐old man with 24 years of T1DM and a recent onset of GS showed an increase in HbA1c from 60 to 72 mmol/mol.^6^ Some case reports of patients with T2DM and GS also report high HbA1c as associated with GS.^15^, ^16^ Poor glycaemic control may impact the risk for GS, and the potential mechanism should be addressed in further studies.

Proliferative retinopathy—which was associated with a lower probability of having GS in our T2DM cohort—is a microvascular diabetes complication and is often present in an asymptomatic state long before diagnosis.^22^, ^23^ This could indicate that a microvascular aetiology of GS is unlikely, but the observed association needs further addressing in mechanistic or histological studies and may be a result of chance. Studying skin biopsies from affected patients and a healthy control group could further contribute to the understanding of the pathophysiology of GS and provide evidence for or against the hypothesis of aberrant reinnervation causing the sweating.

### 4.1 Strengths and limitations

The strengths of our study are primarily the large number of patients included, the access to clinical data, the inclusion of a control group and the relatively high questionnaire response rate, especially in the T1DM cohort. Moreover, the answers to the questions about duration and location of GS add to our knowledge about this diabetes complication. More clinical information is required to make further thorough analyses of autonomic neuropathy, possible sweat as side‐effects of medication, lipid‐profile, duration of symptoms of GS and macrovascular complications.

A study limitation is that the questionnaire has not been validated. There may be a difference between those answering paper questionnaires (DM groups) and those answering online questionnaires (control group). Also, self‐reported information from the control group about diabetes status, kidney status and height and weight may be inaccurate and patients with unclear food triggers were not categorized as having true GS which may have underestimated its prevalence. We also did not have complete retinopathy data for patients with T1DM, and we did not have eGFR data for either group. In the ADA statement on diabetic neuropathy from 2017, GS is limited exclusively to the head and neck region,^2^ while we included sweating from anywhere on the body. Therefore, we may have overestimated the prevalence of GS.

### 4.2 Future aspects

The highly individual perception of the symptoms and varied clinical manifestations of GS suggest an objective clinical classification regarding location, duration, triggers and intensity of sweating to determine types and severity of GS. The classification system could utilize our questionnaire and additional simple methods to quantify sweating, such as the weighing of absorbent papers, as suggested by Dulguerov et al.^24^

Despite the sometimes distressful nature of GS, it is suspected that many people suffering from GS do not bring up symptoms of GS with their physician—and vice versa.^4^ Closing gaps in scientific knowledge as well as creating more awareness among patients and medical care providers about therapy options are key steps in addressing this diabetes complication and a possible unexplored association of GS and diabetic gastroparesis and low heart rate variability should be studied. Our questionnaire needs to be further validated and should include questions about severity of sweating, in both people with DM and in the background population.

## CONFLICT OF INTEREST

None.

## AUTHOR CONTRIBUTIONS

**Carina Kirstine Klarskov:** Conceptualization‐Equal, Data curation‐Lead, Formal analysis‐Lead, Funding acquisition‐Supporting, Investigation‐Lead, Methodology‐Lead, Project administration‐Lead, Resources‐Supporting, Software‐Supporting, Supervision‐Lead, Validation‐Lead, Visualization‐Equal, Writing‐original draft‐Lead, Writing‐review & editing‐Lead. **Elena Von Rhoden:** Conceptualization‐Equal, Data curation‐Equal, Formal analysis‐Equal, Funding acquisition‐Supporting, Investigation‐Equal, Methodology‐Equal, Project administration‐Equal, Resources‐Supporting, Software‐Supporting, Supervision‐Supporting, Validation‐Equal, Visualization‐Equal, Writing‐original draft‐Equal, Writing‐review & editing‐Supporting. **Lise Tarnow:** Conceptualization‐Equal, Data curation‐Equal, Formal analysis‐Supporting, Funding acquisition‐Supporting, Investigation‐Equal, Methodology‐Equal, Project administration‐Supporting, Resources‐Supporting, Software‐Supporting, Supervision‐Equal, Validation‐Equal, Visualization‐Equal, Writing‐original draft‐Supporting, Writing‐review & editing‐Lead. **Birger Thorsteinsson:** Conceptualization‐Equal, Data curation‐Equal, Formal analysis‐Supporting, Funding acquisition‐Supporting, Investigation‐Equal, Methodology‐Equal, Project administration‐Supporting, Resources‐Supporting, Software‐Supporting, Supervision‐Equal, Validation‐Equal, Visualization‐Equal, Writing‐original draft‐Supporting, Writing‐review & editing‐Lead. **Peter Lommer Kristensen:** Conceptualization‐Equal, Data curation‐Lead, Formal analysis‐Lead, Funding acquisition‐Supporting, Investigation‐Lead, Methodology‐Lead, Project administration‐Supporting, Resources‐Supporting, Software‐Supporting, Supervision‐Lead, Validation‐Lead, Visualization‐Equal, Writing‐original draft‐Equal, Writing‐review & editing‐Lead.

## Supporting information

Table S1

## Data Availability

The data that support the findings of this study are available from the corresponding author upon reasonable request.
