# Supplementary material for: Gustatory sweating in people with type 1 and type 2 diabetes mellitus: Prevalence and risk factors
Source: Endocrinol Diabetes Metab. 2021 Aug 10;4(4):e00290. doi: 10.1002/edm2.290 (PMC8502225; doi:10.1002/edm2.290)
Supplement: Supplementary file 1 — Table S1 [file EDM2-4-e00290-s001.docx]

Supplementary table 1: Odds ratio for true gustatory sweating in people with diabetes compared to people without diabetes divided into five age groups.

| **Age group** | **Control group**  **n (case/all)** | **DM group**  **n (case/all)** | **Odds ratio** | **95%CI** | **p** |
| --- | --- | --- | --- | --- | --- |
| 16-29 years | 3/80 | 4/53 | 1.0 | 0.13-7.54 | 1.000 |
| 30-44 years | 10/225 | 15/154 | 2.57 | 0.98-6.76 | 0.056 |
| 45-59 years | 25/523 | 48/348 | 3.75 | 2.14-6.58 | **<0.001** |
| 60-74 years | 16/288 | 45/439 | 1.95 | 1.15-3.31 | **0.015** |
| 75+ years | 0/42 | 17/164 | 4.02 | 1.24-4.13 | **0.009** |
